# Capillary Electrophoresis as a Monitoring Tool for Flow Composition Determination

**DOI:** 10.3390/molecules26164918

**Published:** 2021-08-13

**Authors:** Mihkel Kaljurand, Piret Saar-Reismaa, Merike Vaher, Jelena Gorbatsova, Jekaterina Mazina-Šinkar

**Affiliations:** Faculty of Science, Institute of Chemistry and Biotechnology, Tallinn University of Technology, 19089 Tallinn, Estonia; piret.saar1@taltech.ee (P.S.-R.); merike.vaher@taltech.ee (M.V.); jelenagorbatsova@gmail.com (J.G.); jekaterina.mazina@taltech.ee (J.M.-Š.)

**Keywords:** flow analysis, monitoring, capillary electrophoresis, open source hardware

## Abstract

Flow analysis is the science of performing quantitative analytical chemistry in flowing streams. Because of its efficiency and speed of analysis, capillary electrophoresis (CE) is a prospective method for the monitoring of a flow composition withdrawn from various processes (e.g., occurring in bioreactors, fermentations, enzymatic assays, and microdialysis samples). However, interfacing CE to a various flow of interest requires further study. In this paper, several ingenious approaches on interfacing flow from various chemical or bioprocesses to a capillary electrophoresis instrument are reviewed. Most of these interfaces can be described as computer-controlled autosamplers. Even though most of the described interfaces waste too many samples, many interesting and important applications of the devices are reported. However, the lack of commercially available devices prevents the wide application of CE for flow analysis. On the contrary, this fact opens up a potential avenue for future research in the field of flow sampling by CE.

## 1. Introduction

Flow analysis is the science of performing quantitative analytical chemistry in flowing streams [1]. It can be any liquid material stream withdrawn from a chemical or bioreactor, from a chemical process, or from an environmental source (such as a river or similar source). Although an information about the composition of a particular flow can be obtained by using a set of chemical sensors, those sensors’ lack of the required selectivity and sample processing and separation is an essential part of the flow analysis. This review describes the possibilities of a particular separation method—capillary electrophoresis (CE)—for flow injection analysis (FIA). It reviews up-to-date research in this field. The review is aimed to complement a previous review article [2]. We demonstrate that CE has advantages over other, more popular separation methods such as liquid chromatography (LC)–mass spectrometry, and that the online interfacing of CE is simple in principle but involves complications due to the need for supporting instrumentation. By analyzing some possible examples of published FIA–CE applications for the analysis of various processes, we demonstrate the need for supporting instrumentation results in FIA–CE interfacing, as it is clumsy and unsuitable for miniaturization. This explains why FIA–CE applications are not widespread and many attractive applications are overlooked. In the Conclusion Section are discussions on how to achieve miniaturization and open access in FIA–CE interfacing, and one example of this kind of approach is provided: a monitoring device for checking tap water quality, which can be used in, e.g., citizen science projects.

Separation methods involve the necessary time for separating the sample into components, and flow analysis must consider this time. If the flow composition varies with time (and this variation is usually of interest), then there is certain time interval during which the analysis must be completed to obtain a realistic estimate of the variation course occurring in the flow. We call this interval the time (temporal) resolution of the flow analysis. To provide an idea of the required time resolution durations, some examples are given in Table 1.

For the separation method, the total time required for separation determines the temporal resolution for flow analysis. Comparing CE to LC, the former has several disadvantages over the latter: higher detection limits and being less robust to operate. On the other hand, a smaller consumption of chemicals, being greener, and having better flexibility for miniaturization are the advantages of CE over LC. In the context of this publication, the important advantage is the fact that, in general, the separations in CE take less time than in LC, thus making it more suitable for rapid analysis processes, which require time resolution in the order of seconds to minutes.

## 2. Coupling FIA to CE Online

When the changes in the flow composition are slow compared to the time needed for sample collection, processing, and analysis, the monitoring can be performed offline with any available commercial or homemade instrument based either on the LC or CE method. Online monitoring is needed when the process is analyzed with a short temporal interval, and this requires advanced equipment. In the following, we consider two of the most popular combinations of FIA–CE: direct insertion of a capillary into flow channel and flow gating.

### 2.1. Direct Insertion of a Capillary into the Flow Channel

**Figure 1:**
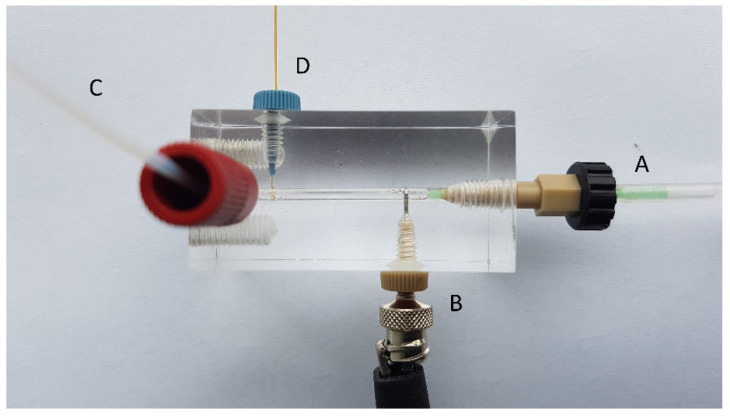
FIA–CE interfacing by direct insertion of a capillary into the flow channel. (**A**) flow in, (**B**) electrode, (**C**) flow out, (**D**) capillary.

The simplest way of interfacing CE to flow is to insert a separation capillary into the flow stream by fixing the capillary inlet end and electrode into the flow channel. This was first demonstrated by Kaljurand et al. [4] and shortly afterward by Kuban et al. [5]. The interface is a quite simple piece of inert plastic (material depends on the chemical composition of the flow) that contains drilled channels for the flow, capillary, and electrode. The interface is usually kept at ground potential to avoid the difficulties of high voltage isolation. An interface schematic is represented in Figure 1.

**Figure 2:**
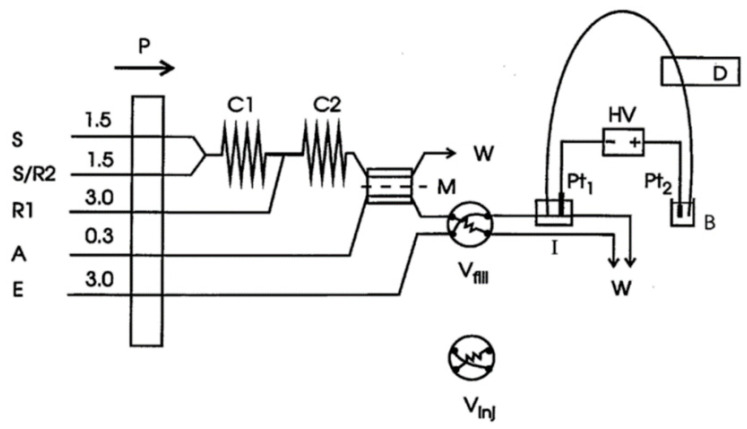
An FIA–CE interface [6]. P is a peristaltic pump, S is the sample flow, R2 is an input for an optional solution, R1 provides the reactant, A is the input for the acceptor solution and E for the electrolyte solution, C1 and C2 are reaction coils, W denotes waste, M is the gas diffusion membrane, Pt_1_ and Pt_2_ are platinum electrodes, HV is a high-voltage supply, D is the detector; I is the FIA–interface, and B is the buffer solution. V_fill_ shows the injector in the fill position and V_inj_ in the inject position. Reprinted with permission from Elsevier.

While the interface itself is simple, the CE analysis process requires supporting equipment for interchanging the sample and the background electrolyte (BGE), for providing liquid flows for rinsing capillaries (such as NaOH or H_2_O), as well as equipment for sample cleaning and processing, since the direct sampling of flow is rarely possible. An example of a set-up that consists of all listed supporting equipment is the publication by Kuban and Karlberg [6]. The equipment was applied to the analysis of real samples such as soft drinks, vinegar, and wine. Besides an FIA–CE interface, it also featured an online gas diffusion membrane separator, which was coupled to a capillary electrophoresis system via the interface (see Figure 2). The sample flow, S, was merged with reactant solutions R1 and R2 at the reaction coils C1 and C2 to transform the analytes of interest into their respective gaseous forms. These transformed gaseous analytes permeated through a polytetrafluoroethylene membrane, M, into an acceptor stream, A. The continuously flowing acceptor stream was led into the interface through a manually operated mechanical valve that performs exchange between the acceptor (sample) and the BGE flow, E. The receiving carrier stream of the sample, a chromate buffer, brought 50 μL of the sample solution to the FIA–CE interface, into which one end of the separation capillary was inserted. As follows from the logic of the set-up, no offline sample pretreatment was required.

**Figure 3:**
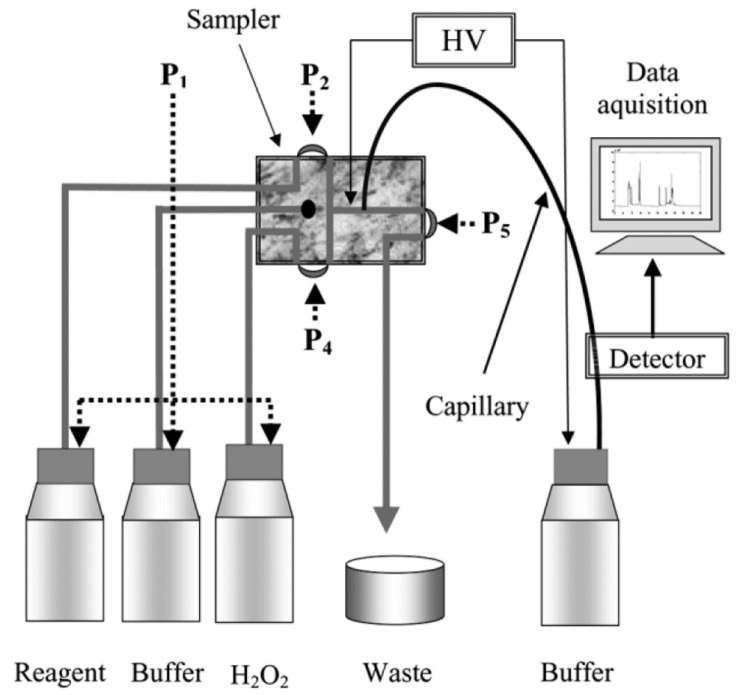
Equipment for monitoring the reaction composition using a pneumatic sampler [7]. Reprinted with permission from Wiley.

Vaher et al. implemented gas exchange pumps for accomplishing complete automatization of the FIA–CE coupling [7] (see Figure 3). In this set-up, the interface body is completed with four polymeric membranes, which can close or open the channels for the sample, P_2_, for the buffer (shown as dot on the interface block) or for the oxidant (H_2_O_2_), P_4_, flow. Pressure P_5_ closes the waste channel. Pressure P_1_ initiates liquid flow in the required channel if the corresponding pressure is released from the membrane. The solenoid valves (not shown in the figure) provide closing pressure to the membranes. Solenoid valves are, in turn, controlled by computer, so complete automatization and programmability of the instrument is established. This equipment was used for the determination of the antioxidative potential of various bioactive phenols found in plant, fruit, and vegetable extracts.

**Figure 4:**
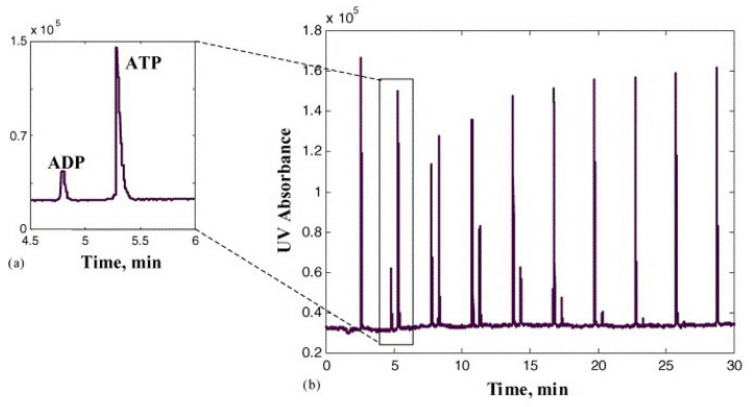
Conversion of ATP to ADP by hexokinase [8]. (**b**) electropherograms recorded during the reaction, (**a**) zoom in on a single electropherogram. Reprinted with permission from Elsevier.

The same FIA–CE interface with membrane valves and gas exchange pumps (controlled by solenoid valves) has been used for studying the flows originating from various bioprocesses. Among these processes are monitoring of the enzymatic conversion of adenosine triphosphate to adenosine diphosphate [8], monitoring of the kinetics of the metabolic conversion of ATP to ADP catalyzed by MgATPases of muscle *Gastrocnemius* skinned fibers [9], monitoring of the degradation of phenols by *Rhodococcus* bacteria [10], and monitoring of the bioaccumulation of Cu, Zn, Co, and Cd by the *Rhodococcus* sp. bacteria (isolated from highly polluted technogenic soil in Estonia) [11]. As an example of these applications, in Figure 4, electropherograms describing the conversion of ATP to ADP by hexokinase are shown. Peaks for the product (ADP) increase, while peaks for the substrate (ATP) decrease [8].

**Figure 5:**
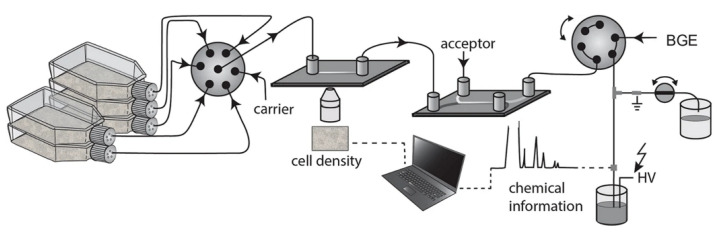
Schematic diagram of the experimental set-up for cell culture monitoring [14].

Breadmore’s group (from the University of Tasmania, Tasmania, Australia) developed an elaborate automated platform for the online, real-time monitoring of suspension cultures based on FIA–CE technology. It integrates microfluidic components for cell counting and filtration with a CE separation technique. This is perhaps the most sophisticated FIA–CE combination reported thus far. Variants of this platform for different applications have been demonstrated in several publications [12,13,14]. For example, in the latter work, equipment for the monitoring of the extracellular content from five parallel suspension cultures was developed. This equipment combines cell density measurements with high-resolution CE separations every 12 min for four days (see Figure 5). The set-up features selector and switching valves, which provide the fluidic control required for sampling from a culture during the analysis of the previous sample from another culture. The sample is sequentially withdrawn from one of the five culture flasks, controlled by the selector valve. To prevent cross-contamination between samples, a carrier solution (5% methanol) is used to rinse the connecting tubing to prevent cross-contamination between samples. Cell density measurements are performed using automated image analysis. The sample is then transported to a microfluidic separator, an H-filter, whose role is to prevent debris from the cell culture entering the capillary. The H-filter extracts analytes into a cell-free solution flow, which is analyzed by CE with a contactless conductivity detector by inserting the inlet end of the separation capillary directly into this flow (as described above in Figure 1).

**Figure 6:**
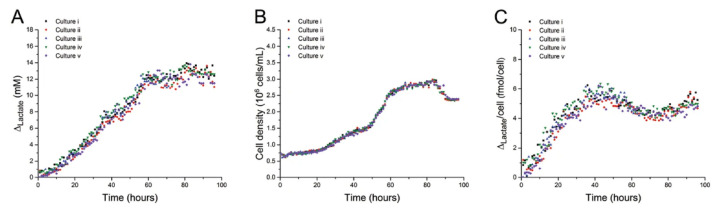
Simultaneous monitoring of five parallel cell cultures of Jurkat cells over four days [14]. (**A**) Changes in lactate concentration, (**B**) changes in cell density, and (**C**) lactate concentrations standardized on cell density.

This system was applied to study the metabolic effects of the drugs rotenone, β-lapachone, and clioquinol using lactate as metabolic indicator. Using appropriate BGE and capillary coating, the authors demonstrated an electropherogram that had well-separated peaks of common anions (chloride, nitrate, and carbonate), lactate, and amino acids glutamine and leucine/isoleucine. In Figure 6, the simultaneous monitoring of five parallel cell cultures of Jurkat cells over four days is represented.

**Figure 7:**
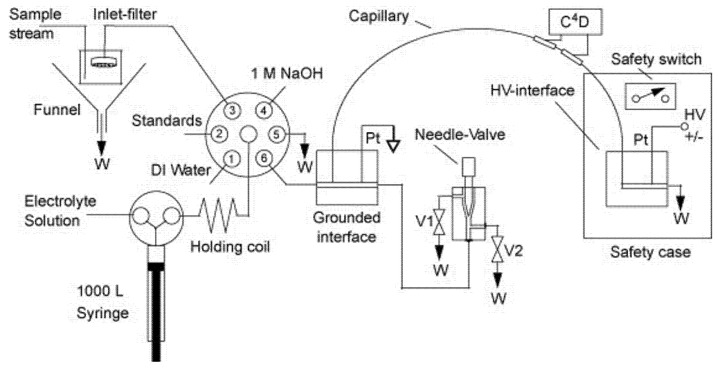
Schematic drawing of the SI–CE–C4D system [15]. C4D, capacitively coupled contactless conductivity detector; HV, high-voltage power supply; W, waste; Pt, platinum electrode; E, electrolyte solution. Reprinted with permission from Elsevier.

**Figure 8:**
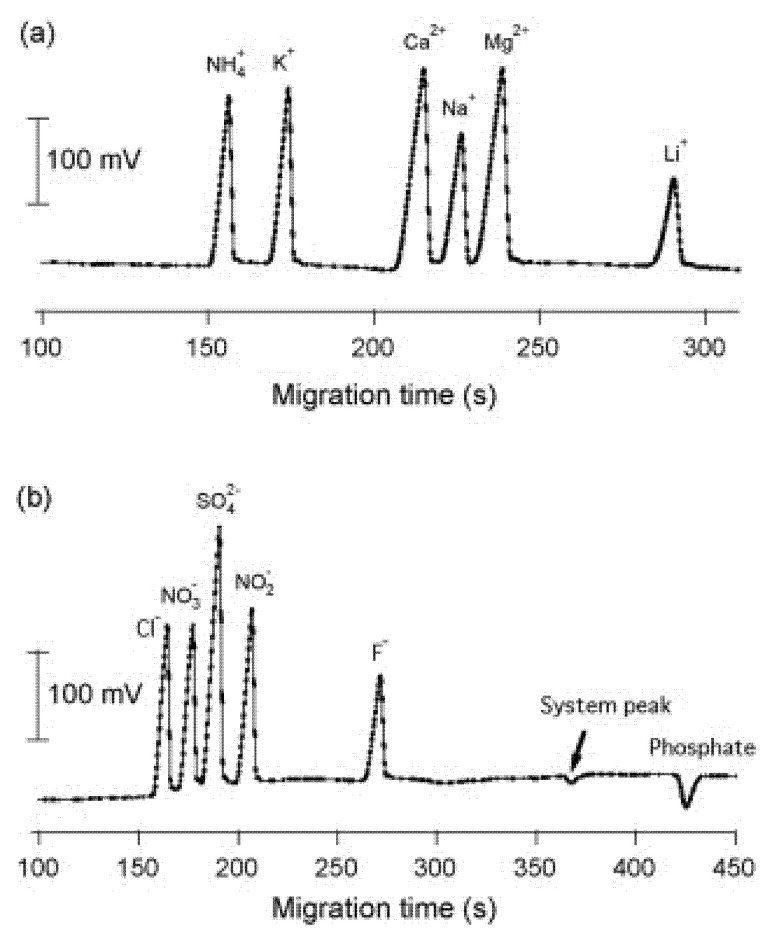
Electropherograms of standard solutions containing either inorganic cations or inorganic anions prepared in deionized water [15]. (**a**) Cations (50 µM), dispensed volume of 40 µL; (**b**) anions (50 µM). BGE: His 12 mM, 18-crown-6 2 mM adjusted to pH 4 with acetic acid. Capillary internal diameter = 50 µm, length l/L = 35 cm/60 cm; high voltage = 20 kV. Reprinted with permission from Elsevier.

Research from Prof. Hauser’s group (Basel, Switzerland) has developed many automatic CE apparatus, which can also be used for online monitoring of the composition of flows. For example, Duc Mai et al. developed sequential injection (SI) manifold analysis for automated monitoring applications based on CE contactless conductivity detection [15]. The set-up (Figure 7), which looks sophisticated at first sight, is rather straightforward. The SI manifold is based on a two-way syringe pump and a multiport valve with a holding coil between the two units. This is used for the supporting operations for CE analysis (initial conditioning of the capillary by flushing with an NaOH solution, rinsing the system with BGE, aspiration of a plug of the sample solution, and passing this volume to the capillary inlet). The hydrodynamically injected volumes into the separation capillary are in the nanoliter range. The sample is injected into the separation tubing by pressurization of the interface while pushing the sample plug past the capillary inlet. An example of the subsequent analysis of a standard mixture of cations and anions at 50 µM is shown in Figure 8.

To evaluate the potential of the instrument for unattended monitoring, it was set up at a pumping station next to the creek Kleine Aa, a tributary to Lake Sempach (in the canton of Lucerne, Switzerland). Analysis of the water from the creek was carried out at intervals of around 35 min for five days during a period of frequent rain (end of April 2009). The concentration curves of the monitored ions essentially displayed random functions. Some of the ions included in the standard mixture could not be found in the natural water system at detectable levels.

**Figure 9:**
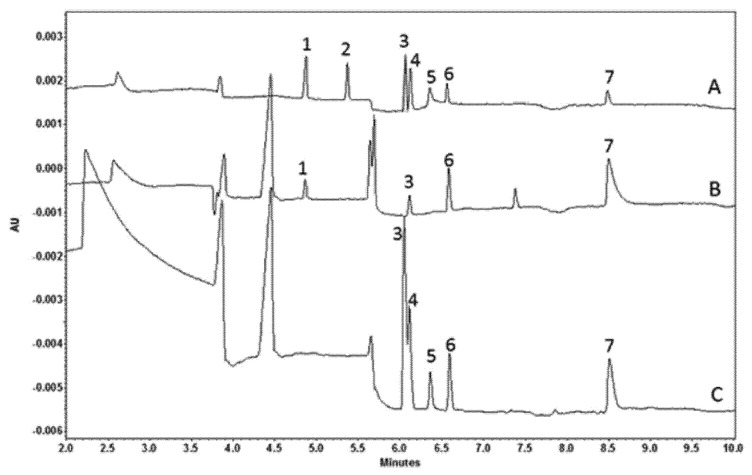
Electropherograms of online analysis of carboxylic acids during a bioreactor cultivation of *K. lactis* [19]. (**A**) Separation of standards at 50 mg/L, (**B**) bioreactor sample at 0 h, and (**C**) bioreactor sample at 60 h. Peak assignments: 1, formate; 2, malate; 3, acetate; 4, glycolate; 5, glyoxylate; 6, PIPES; 7, gluconate. Reprinted with permission from ACS.

In a series of publications, Turkia et al. implemented FIA–CE technology for the monitoring of carboxylic acid production by *Gluconobacter oxydans* [16], the amino acid uptake by yeast during beer fermentation [17], and the phenolic compound production in the hydrolysates of lignocellulosic biomass [18]. The monitoring in these works was performed offline. More interesting is a publication [19] where the authors describe how a commercial CE instrument (Beckman Coulter) was skillfully modified to achieve online monitoring capability and automatization of the full monitoring process. In this work, using a peristaltic pump, the sample was withdrawn from the bioreactor and delivered for filtration and analysis into the flow-through sample vial. The vial was made from a solid PEEK block by drilling and inserting inside a plastic vial holder of the Beckman Coulter CE instrument. The sample vial was placed in the inlet side of the buffer tray in the CE instrument, from where the sample was injected. A second peristaltic pump was used for withdrawing the sample from the sample vial to the waste. To insert the tubing from the pump to the sample vial, a hole was drilled into the cover of the CE device. The equipment was used to monitor the carboxylic acid production by *Kluyveromyces lactis* and *Saccharomyces cerevisiae* across six days of automatic and continuous operation with an analysis time (time resolution) of less than 20 min. When the production of metabolites of cultivation appeared to be monotonically rising/falling, the electropherograms were more interesting, demonstrating that the authors had developed successful protocols for the separation of cultivation products (see Figure 9 as an example).

### 2.2. Flow Gating Interfaces

**Figure 10:**
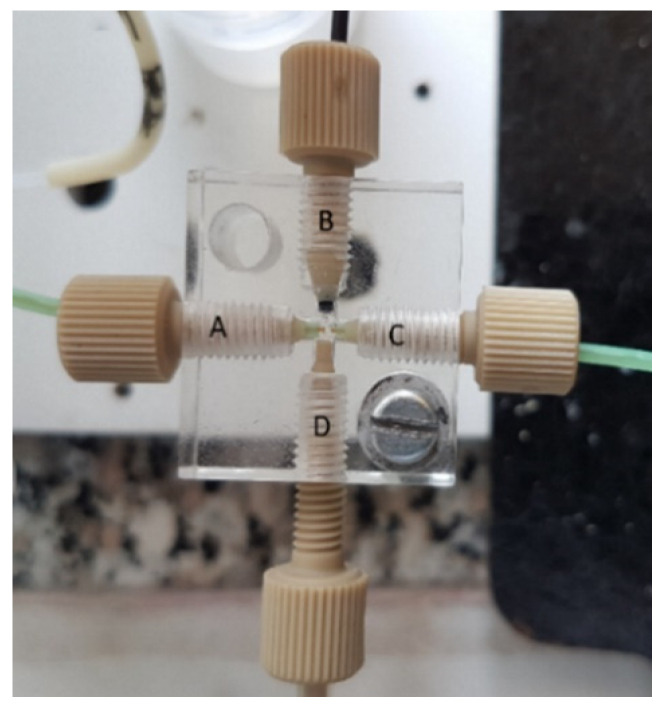
Photograph of a flow gating interface (photo from the authors’ laboratory). A—sample capillary, B—BGE flow in, C—separation capillary, D—BGE flow out.

A flow gating (FG) interface is, in principle, a cross-connection of four flow lines (see Figure 10). A separation capillary, C, is inserted into the perpendicular continuous flow of a BGE (channels B and D). A sample capillary is fixed in line with the separation capillary through the cross arm, A. For sampling, the BGE flow is stopped, and a sample is forced to flow through the sample capillary into the gap between the sample and separation capillaries. Electrokinetic sampling then takes place, and if the sample flow from the capillary is stopped and the BGE flow is resumed, the normal CE separation process begins.

The sampler was first proposed by Hooker and Jorgenson and they used the name “flow gating” (FG) for this type of interface [20]. Although the interface looks extremely simple, it obviously needs supporting instrumentation to operate. There must be a computer-controlled pump for providing the BGE to the cross. In addition, there must be the possibility for automatic activation of the sample flow through the corresponding capillary. Moreover, both capillaries must be precisely aligned, and the width of the gap between the capillaries is critical. All of this makes the interface rather clumsy, fragile, and unstable to operate. This is perhaps the reason why this interface is rarely used. Some remarkable examples are the sheath FG interface for the online coupling of solid-phase extraction with CE [21], and the PDMS interface for FG and reagent mixing in CE [22].

**Figure 11:**
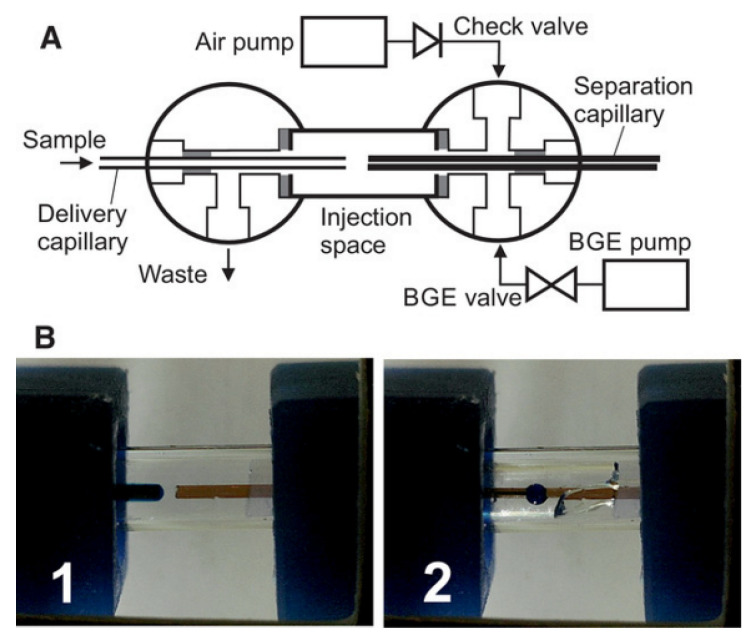
An air-assisted flow gating interface for CE [23]. (**A**) A schematic drawing of the interface; (**B**) a photograph of the flow gating interface showing: 1, flushing off the BGE from the gap between the capillaries; 2, introducing a sample droplet. Reprinted with permission from Wiley.

Recently, Opekar and Tůma introduced a notable innovation into the construction of FG interfaces [23]. In their design (see Figure 11), the BGE is flushed off from the gap between the capillaries before sampling, and a sample is introduced as a droplet before the separation capillary. In this way, the mixing of a sample with BGE is avoided. Although rather intricate in construction, the authors were able to obtain nice electropherograms of cations with this set-up.

## 3. Open Access CE Instrumentation for Monitoring

As follows from the above, the use of CE for the online analysis of flows requires usually specific instrumentation, and to complete this in a laboratory is frequently beyond the capabilities of a typical analytical chemist. Here, a helpful new trend in various fields in measurement science (including analytical chemistry) is becoming steadily popular. This trend, known as “open source hardware”, could be of great help to those individuals who are willing to prepare their own FIA–CE instrumentation. The Open Source Hardware Association defines it as such: “Open source hardware is hardware whose design is made publicly available so that anyone can study, modify, distribute, make, and sell the design or hardware based on that design.” Ideally, open source hardware uses readily available components and materials, standard processes, open infrastructure, unrestricted content, and open source design tools to maximize the ability of individuals to make and use hardware [24]. This new paradigm is becoming widely accepted in scientific communities, and open source hardware is finding its steady place in chemistry research. There are several publications of general interest, which describe how to implement open source hardware in chemical research [25,26]. For CE, particularly, Kuban et al. provided the most up-to-date information on open source hardware and software resources, enabling the construction and utilization of an open source CE instrument. They demonstrated that the unique flexibility, low cost, and high efficiency of CE makes it particularly suitable for open source instrumental development [27]. They provided an overview of hardware and software sources, with emphasis on the availability of open source information on the web and in the scientific literature. Hauser’s group reported several successful open source set-ups composed of commercially available parts without the requirement of mechanical and electronic workshop facilities [28,29]. To make the fabrication of CE instruments especially easy for interested persons, the latter publication even includes a “shopping list “of the needed parts, together with vendors.

**Figure 12:**
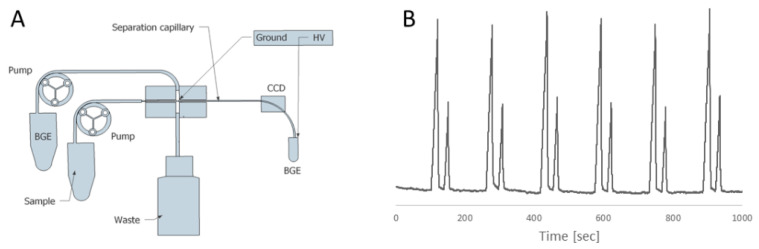
An example of a simple FIA–CE interface. (**A**)—experimental set-up. (**B**)—set of electopherograms of anions of Tallinn (Estonia) tap water (recorded by switching of the peristaltic pumps). Peaks: first, Cl^−^ and second, SO_4_^2−^. Electrophoretic conditions: BGE- 1M acetic acid, HV = 10 kV, capillary: 20 cm length and 75 µm internal diameter.

For demonstration purposes and to acquire knowledge about the possible problems that a prospective person could encounter by composing their own FIA–CE instrumentation, the authors of this publication completed the simplest possible FIA–CE instrumentation consisting, as much as possible, of commercially available parts. A schematic of the instrument is shown in Figure 12. It consists of two micro-peristaltic pumps (RP-Q1 Series by Takasago, Nagoya, Japan [30]), which provide sample and BGE solutions to the flow gating cross (made in a local workshop, but can be replaced by an Upchurch PEEK cross). A high-voltage power supply (DX 250, EMCO, Sutter Creek, CA, USA) provides the needed separation voltage. A contactless conductivity detector was built in house by the open source description provided by do Lago [31]. The set-up was used for successful monitoring of the contents of the cations in tap water using acetic acid BGE. However, we found that the flow gating sampler is not robust enough. Its optimal performance depends critically on the width of the gap between sample and separation capillaries. In addition, the need for continuous supply of the BGE is in contrast with one of the main advantages of CE: low consumption of chemicals. In experiments described in Figure 12, the discharge rate of the micro peristaltic pump (Takasago miniature peristaltic pump RP-Q1.2N-P20A-DC3V) was 0.2 mL/min. This means that during one-hour run the sampler consumes about 12 mL BGE.

It is obvious that some experience in CE technology will help to complete even such trivial set up. The cost of the parts is low, except for the contactless conductivity detector. For us, the detector seems to be the main obstacle in achieving the goals of building open source FIA–CE instrumentation. Although one has a very detailed description and instructions on how to build an open source CCD [31], it is still difficult to imagine that a person not experienced in electronics could complete this task. For using optical detectors, we have a similar problem: Detailed open source descriptions are available (see, e.g., [32]), but here, competence in optics would be useful. Therefore, the development of a cheap and simple detector for CE is urgently required for achieving the goals of open source hardware. For example, a similar problem seems to have found a solution in gas chromatography. Metal oxide semiconductor (MOS) gas sensor detectors appear to be cheap detectors for gas analysis. The appearance of gas sensors has made possible the building of an extremely simple and robust yet still powerful gas chromatograph, which can be built by a layman and which would be useful for citizen science [33].

## 4. Conclusions

As follows from the discussion above, many possible applications of flow monitoring by CE have been reported, but the number of examples is still few. This is because no monitoring instrumentation is commercially available. All of the samplers that are currently used for online monitoring are purpose-made at the user’s laboratory. They tend to be of an overly elaborate construction, which makes it difficult for interested persons to copy them. Finally, samplers tend to waste samples and BGE.

Still, the FIA–CE combination for the monitoring of flow has some advantages, which justifies further research to overcome the present obstacles. CE is the only analytical separation method that is amenable for miniaturization. If the supporting equipment can be reduced correspondingly, the appearance of separation-based sensors can be expected. These sensors could then be used online for many attractive applications, with bioreactor monitoring in situ being one of the obvious applications. This includes designing miniaturized bioreactors and cell culture systems in vitro and monitoring nutrients and metabolic products. Moreover, neurochemistry or drug metabolism and behaviors in awake, freely roaming animals could be monitored. Even direct telemetric control of such animals could be considered: observing the natural behavior of an animal and correlating it with biochemical events in the brain.

Finally, we wish to point out one particularly attractive application. If miniaturization appears to be successful, such FIA–CE combinations could be useful, for example, for space applications. Bioprocess studies under microgravity environments are being performed presently in the International Space Station, which makes such studies extremely expensive. On the contrary, miniaturized FIA–CE systems could be accommodated in nanosatellites for study, for example, of the effects of microgravity on various cellular systems. Placing FIA–CE sensors on nanosatellites would make such studies much cheaper and, therefore, available to a much wider group of interested researchers (including graduate students) than is possible now.

## Figures and Tables

**Table 1:** The temporal resolution necessary for various applications (from [3]).

Application/Compound	Temporal Resolution
Neurotransmitters	Milliseconds to seconds
Drug transport and metabolism	Minutes to hours
Energy biomarkers (e.g., glucose and lactate)	Minutes to hours
Peptides	Minutes
Bioreactor monitoring	Minutes to hours
Reactive oxygen and nitrogen species	Minutes
Antioxidants (e.g., glutathione and ascorbic acid)	Minutes to hours
Environmental monitoring	Hours to days
